# Feasibility and preliminary efficacy of a skill-based lifestyle intervention for enhancing cooking abilities and physical fitness in young adults with intellectual disabilities

**DOI:** 10.1016/j.dhjo.2024.101767

**Published:** 2024-12-04

**Authors:** Jessica C. Danon, Lyndsie Koon, Joseph R. Sherman, Anna M. Rice, Scott Quaife, Brian C. Helsel, Amy Bodde, Lauren T. Ptomey

**Affiliations:** a Department of Internal Medicine, The University of Kansas Medical Center, Mail Stop 1007, 3901 Rainbow Blvd, Kansas City, KS, 66160, USA; b Research and Training Center on Independent Living, University of Kansas, 100 Sunnyside Ave, Lawrence, KS, 66045, USA; c Down Syndrome Innovations, 5916 Dearborn Street, Mission, KS, 66202, USA; d Department of Neurology, The University of Kansas Medical Center, 4350 Shawnee Mission Parkway, Mailstop 6002, Fairway, KS, 66205, USA

**Keywords:** Physical activity, Intellectual disability, Down syndrome, Meal preparation, Cooking, Fitness, Nutrition

## Abstract

**Background:**

Individuals with intellectual disabilities (ID) often experience poorer diet quality and lower physical fitness levels as they transition from adolescence to adulthood.

**Objective:**

The purpose of this study was to assess the initial feasibility and efficacy of Chef-ID, a 12-week intervention designed to improve cooking skills and physical function in young adults with ID.

**Methods:**

Young adults with ID attended weekly group sessions which provided hands-on cooking skills, nutrition education, and exercise. Participants were also asked to attend monthly, virtual, goal setting sessions. Feasibility outcomes included attendance, retention, and safety. Preliminary efficacy outcomes included cooking skills, lower body muscle strength, grip strength, aerobic capacity, and body weight. Paired t-tests were used to assess the differences in cooking skills, strength measures, aerobic capacity, and weight after the 12-week intervention.

**Results:**

Study retention was 95 %, attendance exceeded 85 % for all sessions, and no serious adverse events were reported. The number of cooking skills participants could do independently (p = 0.005), the number of cooking skills requiring only a verbal prompt (p = 0.01) and lower body strength (p = 0.004) significantly improved across the 12-week intervention. The number of cooking skills participants had no exposure to (p = 0.01) and weight (p = 0.036) significantly decreased across the intervention. No significant changes were observed for upper body strength or aerobic capacity.

**Conclusions:**

The Chef-ID intervention was feasible with desirable initial effects on cooking skill independence, exposure to cooking skills, lower body strength, and weight. The Chef-ID intervention holds promise in enhancing cooking skills and physical function among young adults with ID.

**Trial registration number:**

Clinicaltrials.gov, NCT05385016.

## 1. Introduction

Intellectual disability (ID) is defined as a developmental disability identified prior to age 22 characterized by significant limitations in both intellectual functioning (IQ ≤ 75) and adaptive behaviors.^1^ ID co-occurs or is fully comorbid with other developmental disabilities including, but not limited to, autism spectrum disorder (ASD) and Down syndrome (DS).^1^ The prevalence of obesity (body mass index [BMI] ≥ 30 kg/m^2^) and cardiometabolic diseases are higher in individuals with ID compared with the general population,^2–4^ in part due to low levels of physical activity and poor diet quality.^5–7^ However, there is a paucity of interventions focused on weight management, promotion of physical activity, or improvement of diet intake in individuals with ID.

The period between adolescence and adulthood, i.e., young adulthood, holds particular significance for individuals with ID.^8,9^ During this period adolescents no longer have the daily structure of attending public school with access to specialized services such as physical and occupational therapy and may spend more time at home or residential day centers, begin to live independent of their parents, or work outside the home, contributing to increased consumption of higher calorie processed foods and decreased physical activity (PA) leading to accelerated weight gain.^10–12^ Thus, the transition period may be an opportune time to employ interventions that focus on improving dietary intake and physical activity.

Data from young adults without ID suggests that higher levels of food preparation skills are associated with reduced use of fast-food and an increased likelihood of meeting dietary objectives for fat, calcium, fruit and vegetable and whole-grain consumption.^13^ Adequate cooking skills during young adulthood were associated with more frequent preparation of meals including vegetables and less frequent consumption of fast food when analyzed 10 years. later.^14^ Similarly, individuals without ID see a 13–17 % decline in moderate to vigorous physical activity between 16 and 20 years of age.^15^ Previous research in young adults without ID suggest that participation in exercise can promote PA habit formation, encourage PA participation and maintenance,^16,17^ improve physical fitness,^18,19^ and may reduce cardiometabolic disease risk and mental health problems.^20,21^ However, given the cognitive and physical disparities between individuals with and without ID, it is unknown if similar interventions could yield comparable outcomes in young adults with ID.

Reports in the literature suggest that acquiring skills related to healthy eating, meal preparation and kitchen safety are seen as important for individuals with ID and their caregivers.^22–25^ For example, Subach and Sullivan,^22^ examined interviews with eight young adults with ID (age ~26 years) and their caregivers who participated in a 12-week culinary skills intervention and reported that participants were interested in being independent with meal preparation and could prepare simple meals independently, and caregivers reported that independent meal preparation was achievable with adequate training. Additionally, intake data from 337 adolescents/young adult patients at a hospital-based Down syndrome (DS) clinic noted patients ranked learning about healthy foods and preparing meals as the 1st and 4th life skills of greatest importance to learn right now; however, less than 10 % of adolescent/young adults indicated they could prepare their own meals with 4 % of adults with DS reporting they were able to cook independently.^23^

There is limited research to support the feasibility of conducting cooking skill interventions in individuals with ID. However, the previous studies collected qualitative data only,^22,26^ did not meet feasibility and retention goals,^27^ or were only conducted in older adults with ID^28^ or individuals with ASD.^29–32^ Additionally, none of the studies included an exercise component with a focus on improving physical function. To fill this gap we developed an intervention, called Chef-ID, to improve both cooking skills and physical function in young adults with ID. The purpose of this paper is to report on the initial feasibility and efficacy of the Chef-ID intervention.

## 2. Methods

### 2.1. Overview

This trial, which was approved by the University’s Institutional Review Board, was conducted in the United States from May 2022 to October 2023. Over the course of 18 months, 4 cohorts of young adults (18–30 years of age) enrolled in a 12-week single arm pilot trial of the Chef-ID intervention. The Chef-ID intervention consisted of weekly, 2 h in-person, group sessions (~5–6 young adults, 2 interventionists/group) that included 60-min of hands-on cooking skills/food preparation, 15-min of nutrition education, and 45-min of exercise focused on physical function, as well as monthly individual 20-min remotely delivered (Zoom^®^) support sessions for the young adults and their caregiver. The primary aim was to assess feasibility (retention, adherence, safety) along with changes in cooking skills, outcomes associated with physical function (upper and lower body strength, aerobic capacity), and body weight across the 12-week intervention.

### 2.2. Participants

Participants were young adults (18–30 years of age), with caregiver reported mild to moderate ID. Additional inclusion criteria included: sufficient functional ability to communicate through spoken language; internet access in the home; transportation to and from the study site; ability to participate in small group environment safely per caregiver report; and a caregiver who agreed to serve as a support partner in the study. Participants were excluded if they had a serious medical risk, such as cancer or a recent cardiac event, or were unable to participate in moderate to vigorous physical activity (MVPA). Cohorts of 5–6 participants (n = 4) were recruited through community organizations as well as social media and word of mouth. Written informed consent was obtained from either the participant (self as guardian) or a legal guardian with participant assent.

### 2.3. Intervention

#### Overview.

In-person group sessions were conducted at 1 of 2 locations, one in a metropolitan city and the other in a mid-sized college town in a rural area of the United States. Each location contained a teaching kitchen which included 4-5 separate cooking stations, e.g., oven/stove/counter space, and small appliances and cooking utensils available in a typical kitchen, plus an open space for performing exercise. Sessions were led by an occupational therapist and registered dietitian (cooking/nutrition education sessions) and an exercise physiologist or personal trainer (exercise sessions) with the assistance of a trained interventionist.

#### Cooking skills/nutrition education.

The content and progression of the cooking skill education sessions was developed based on the “Active Engagement” framework proposed by Goldschmidt et al.^33,34^ This stepwise program was designed to promote participant autonomy, competence, relatedness, and preference and improve basic cooking skills to improve independence in the kitchen environment for adults with ID. Interventionists demonstrated the skills and participants worked, individually or in pairs, to master the selected skills needed to prepare and clean up for a snack or a small meal. Nutrition education adapted from the HealthMatters^™^ curriculum^35^ complemented that week’s recipe and was delivered during the group class. Table 1 provides a full rubric of the cooking skills, food safety and nutrition education covered in the intervention. Adaptive kitchen utensils and tools were available for participants requiring accommodations and visual recipes were provided to breakdown each skill needed to complete each step of the recipe. All recipes were easily modifiable for participants with celiac disease (gluten free) and/or lactose intolerance (dairy free).

#### Physical function training.

Group-based exercise sessions were designed to focus on constantly varied, multi-joint movements with increasing intensity based on individual ability and tolerance intended to mimic movements associated with physical function in daily life e.g., squats, overhead press, deadlift, burpees, weighted ropes, medicine balls etc. Sessions rotated between 3 types of workouts across the intervention: 1) the completion of as many rounds as possible of a 3–4 exercise circuit within a specified time frame (10–15 min), 2) the completion of a circuit of 3–4 exercises in the shortest amount of time possible, 3) repeated completion of a series of 1 min bouts of 4–5 different exercises and a 1-min. rest period over a specified time frame, e.g. 12 min. Exercises were scaled to increase or decrease difficulty level based on participant skill.

#### Individual SupportSessions.

Once a month the participant and their caregiver met with one interventionist via Zoom^®^ video conferencing for approximately 20 min to discuss progress in the group classes and provide individual support related to nutrition and physical activity. These sessions provided education for the participant and caregiver to implement the skills learned in class into the home setting. Interventionists shared adaptive techniques from the cooking class (use of color-coded measuring cups to portion food, tips on safe/adaptive knife use, etc.) as well as provided home-based exercise strategies.

## 3. Outcomes

All efficacy outcomes were assessed at our on-campus laboratories at baseline and 12 weeks by trained staff with a master’s degree in kinesiology who were separate from the interventionist.

### 3.1. Feasibility outcomes

#### Attendance.

Attendance at virtual support sessions and in-person group sessions, i.e., the percentage of sessions attended, was assessed from interventionist records.

#### Retention.

Retention was measured as percent of participants retained throughout the intervention.

#### Safety.

Safety was determined by number and type of serious adverse events.

### 3.2. Preliminary efficacy

#### Cooking skills.

Participant’s independence level with cooking skills were assessed using cooking and kitchen domain items in the Assessment of Functional Living Skills (AFLS): Independent Living Skills.^36^ The AFLS is routinely used by applied behavioral analysts working with individuals with ID to track the development of essential skills for achieving independence. Parents/caregivers were asked to complete the survey on behalf of the participant, and were provided with 48 different cooking skills and asked 1) if the participant has had the opportunity to attempt the skills before (Yes/No), and 2) If yes, the level of independence needed to complete the skills, e.g. independently with a visual prompt, with a verbal prompt, with a modeling prompt, with physical guidance. For this analysis we assessed changes in the total number of skills participants could do independently and with only a verbal prompt.

#### Physical outcomes.

Lower body muscle strength was assessed using a standard 5-repetition maximum protocol^37^ on a Cybex plate-loaded leg press. Grip strength of the dominant and non-dominant hands was assessed using a Jamar hand grip dynamometer (JWL Instruments, Chicago, IL) and was used as an indicator of upper body strength. Participants were asked to stand straight with their elbows flexed at 90° and instructed to exert as much force as possible on the dynamometer. Three measures were collected from both their dominant and non-dominant hand, and scores from each hand were averaged separately. Aerobic capacity was assessed using the 2-Minute Step Test, which has previously been used in individuals with ID.^38,39^ Participants were instructed to march in place by alternating raising their knees to a level halfway between the patella and top of the iliac crest as many times as possible in the 2 min. The number of times the right knee reached the mark was recorded.

#### Anthropometrics.

Participants were weighed in kilograms (kg) on a calibrated scale (Model #PS6600, Belfour, Saukville, WI) to the nearest 0.1 kg. Height was assessed in centimeters (cm) using a portable stadiometer (Model #IP0955, Invicta Plastics LTD, UK) to the nearest 0.1 cm. Weight and height were measured in duplicate with the average measures used to calculate BMI (kg/m^2^).

## 4. Analysis

Demographics, as well as feasibility and efficacy outcomes, are presented as means ± standard deviation for continuous measures and frequency (percentage) for categorical variables. Outcome measures were checked for normality using histograms, Q-Q plots, and Shapiro-Wilk tests. Paired t-tests were used to assess the differences in cooking skills, strength, aerobic capacity, and weight after the 12-week intervention. Statistical significance was set at p < 0.05 and R (version 4.2.2)^40^ was used for this analysis.

## 5. Results

Fifty young adults with ID were assessed for eligibility, and five were found to be ineligible due to exceeding the age limit (n = 3), not being able to communicate through spoken language (n = 1), not having a caregiver who could serve as a study partner (n = 1). Twenty-four eligible participants either had time conflicts or had expressed interest after the study was discontinued. Twenty-one young adults with ID enrolled in the intervention. Participants were ~22 years of age, 65 % females, 48 % non-Hispanic white, and had a mean BMI of 29 kg/m^2^. Almost all participants (91 %) lived at home with a parent. Full baseline characteristics are presented in Table 2.

### Feasibility.

Study retention was 95 % at 12-weeks with one participant withdrawing at week 6 due to time conflicts. No serious adverse events occurred during the intervention. Participants attended 89 % of the cooking sessions, 87 % of the physical function sessions, and 94 % of the monthly Zoom^®^ support sessions.

### Initial Efficacy.

Baseline and post intervention values for cooking skills, upper and lower body strength, aerobic capacity, and body weight for the twenty completers are presented in Table 3. The number of cooking skills participants could do independently (+2.8 skills, p = 0.005) or with only a verbal prompt (+6.2 skills, p = 0.01) and lower body strength (+25lb, *p* = 0.004) significantly improved across the 12-week intervention. The number of cooking skills participants had never been exposed to (−4.9 skills, p = 0.01) and body weight (−1.1 kg, p = 0.036) significantly decreased across the intervention. No significant changes were noted for upper body strength or aerobic capacity.

## 6. Discussion

The Chef-ID intervention was feasible in young adults with ID. Additionally, the intervention demonstrated initial efficacy in exposing young adults with ID to new cooking skills, increasing their level of independence in the kitchen and increasing lower body strength, as well as decreasing body weight.

Participants were able to perform ~3 additional cooking skills completely independently, and 6 additional skills with only verbal prompts after the completion of the intervention. Cooking skills that improved the most included safely slicing foods with a sharp knife, bringing stovetop burners down from a boil to a simmer, and measuring out liquid ingredients. Statistically significant weight loss (−1.03 ± 2.04 kg or ~2 %) was observed after the intervention. However, it is unknown what variables accounted for the weight loss. Future trials should assess dietary intake and the impact of an intervention promoting cooking skills on weight loss.

Previous research demonstrates that young adults with ID exhibit diminished strength and aerobic capacity in comparison to their peers without ID.^41,42^ These physical function skills serve as crucial predictors of daily living activities, employment prospects, and overall independence in adulthood for individuals with ID.^43–45^ However, there is a scarcity of interventions specifically targeting physical function for young adults with ID. The results of the current pilot trial observed improvements in lower body strength, however, no changes in aerobic capacity or upper body strength were noted. Though we did not collect data on physical activity at home, this result may be attributed to insufficient or noncompliance of the home exercise strategies or to the low frequency of the in-person exercise sessions (once a week).Future research should consider measuring daily physical activity, and increasing the frequency of exercise sessions or providing more guidance for home-based exercise training to supplement weekly sessions.

To our knowledge this is the first intervention designed to improve both cooking skills and physical function in young adults with ID. Additional study strengths include the use of an evidence-based framework for teaching cooking skills to individuals with ID, collection of outcomes by trained staff familiar with working with the population and an ethnically and racially diverse sample. Study weaknesses include the lack of information about socio-economic status and level of rurality. Additionally, most participants lived at home with a parent, and all participants had access to transportation to the study site limiting the generalizability of the intervention. Additionally, this was an unpowered pilot trial, lacking the necessary statistical strength and control group to detect meaningful changes over time. Furthermore, the use of a pre-post design without long-term follow-up restricts our understanding of sustained intervention effects.

## 7. Conclusion

The Chef-ID intervention was feasible with desirable initial effects on cooking skill independence, exposure to cooking skills, lower body strength, and weight. The Chef-ID intervention holds promise in enhancing cooking skills and physical function among young adults with ID. Adequately powered randomized controlled trials that include a follow up period or maintenance measurements after intervention are needed to confirm these preliminary findings and inform future interventions targeting this population.

## Figures and Tables

**Table 1:** Chef-ID cooking skills lesson rubric.

Week #	Active Engagement Program Stages	Recipe/Lesson	Description	Nutrition Lesson	Cooking Skills addressed
Wk. 1	^a^Stage 0: Mix, Measure stir	Energy balls	Energy Balls Class: Participants learn basic cooking skills and technique (e.g. mixing, measuring) and examine sensory aspects of food (color, texture) in a peer-to-peer social setting. Staff supported with adaptive equipment, managing safety and attention to task.	Introduce healthy habits and food groups.	- Stir separated items into a mixing dish - Measures solid volume - Stirs items in a mixing bowl - Uses timers for food - Clean up and food storage
Wk. 2	Stage 1: Learning to Chop, Cut, Peel, Grate	Salad production	Vegetable Salad Class: Participants learn kitchen knife safety and practice slicing, chopping with knife or adaptive equipment, peeling and grating in a peer-to-peer social setting. Participants execute choice and preference over salad ingredients. Staff supported with adaptive equipment, managing safety and attention to task.	Introduce Myplate and vegetables/knife skills.	- Safely slices with sharp knife (e.g. slices vegetables) - Washes fruits and vegetables - Makes a salad - Clean up and food storage
Wk. 3	Stage 2: Introduction to Small Appliance Cooking	Avocado toast/PB banana toast	Simple Recipe/Snack Class: Participants learn how to use a toaster oven safely to make a simple warm snack in a peer-to-peer social setting. Participants execute choice and preference over toast toppings and carry over measuring and mixing from Stage 0 and knife skills from Stage 1. Staff supported with adaptive equipment, managing safety and attention to task.	Myplate fruit and grains and balanced snacks	- Uses toaster - Puts leftovers away correctly (appropriate dished and wrapped/has lid) - Clean up and food storage
Wk. 4	Stage 2: Introduction to Small Appliance Cooking	Smoothie	Simple Recipe/Snack Class: Participants learn how to use a blender safely to make a smoothie in a peer-to-peer social setting. Participants execute choice and preference with fruit toppings and carry over measuring and mixing from Stage 0 and knife skills from Stage 1. Staff supported with adaptive equipment, managing safety and attention to task.	Myplate protein and dairy and balanced meals	- Uses blender - Measures liquid volume - Washes fruits and vegetables - Clean up and food storage
Wk. 5	Stage 3: Introduction to Large Appliance Cooking	Eggs scramble on stove	Meal Production: Participants learn stove safety in a peer-to-peer social setting. Emphasis on independence with safely using large kitchen appliance, raw food handling. Participants execute choice and preference over vegetable additions. Carry over measuring and mixing from Stage 0 and knife skills from Stage 1. Staff supported with adaptive equipment, managing safety and attention to task.	Importance of breakfast and raw food safety	- Safely handles eggs, meat, seafood (refrigeration of foods, cleaning of cutting materials) - Cooks at least 2 types of eggs (scrambled, fried, omelet, hardboiled) - Identifies when a stovetop is hot - Adjusts stove burners - Uses spatula - Cleans greasy pans - Clean up and food storage
Wk. 6	Stage 3: Introduction to Large Appliance Cooking	Pasta salad	Meal Production: Participants continue to practice stove safety in a peer-to-peer social setting. Emphasis on independence with safely using large kitchen appliance. Participants execute choice and preference over vegetable additions. Carry over measuring and mixing from Stage 0 and knife skills from Stage 1. Staff supported with adaptive equipment, managing safety and attention to task.	Portion control	- Uses colander - Identify when a stovetop is hot - Puts leftovers away correctly (appropriate dished and wrapped/has lid) - Boils water - Adjusts stove burners - Adjust from boiling to simmering - Makes pasta - Clean up and food storage
Wk. 7	Stage 2 and 3: Small and Large Appliance Cooking	Black bean brownies	Meal Production: Participants learn oven safety and review small appliance (blender) safety in a peer-to-peer social setting. Participants execute choice and preference on fruit additions. Emphasis on independence with safely using kitchen appliances. Carry over measuring and mixing from Stage 0 and knife skills from Stage 1. Staff supported with adaptive equipment, managing safety and attention to task.	Mindful eating/cravings	- Uses colander - Uses blender - Determines when non-meat foods are fully cooked (e.g., temp, time, softness) - Measures liquid volume - Puts leftovers away correctly (appropriate dished and wrapped/has lid) - Stirs items in a mixing bowl - Uses oven mitts for hot items - Places hot pots on pot rests - Clean up and food storage
Wk. 8	Stage 2 and 3: Small and Large Appliance Cooking	Broccoli mac n cheese	Meal Production: Participants continue to practice stove safety and introduce small appliance (microwave) safety in a peer-to-peer social setting. Emphasis on independence with safely using kitchen appliances. Participants execute choice and preference on vegetable additions. Carry over measuring and mixing from Stage 0 and knife skills from Stage 1. Staff supported with adaptive equipment, managing safety and attention to task.	Healthy substitutions/food labels	- Thaws frozen foods (via sink, counter, refrigerator, and/or microwave) - Puts leftovers away correctly (appropriate dished and wrapped/has lid) - Uses colander - Identifies when a stovetop is hot - Uses microwave & can safely use a microwave (can put things in/take things out) - Boils water - Adjusts stove burners - Adjusts from boiling to simmering - Makes pasta - Clean up and food storage
Wk. 9	Stage 3: Large Appliance Cooking	Fish taco	Meal Production: Participants review oven safety in a peer-to-peer social setting. Emphasis on independence with safely using large kitchen appliance, raw food handling. Attention on participant choice with toppings and preferred assembly of dish. Carry over measuring and mixing from Stage 0 and knife skills from Stage 1. Staff supported with adaptive equipment, managing safety and attention to task.	Healthy eating to prevent chronic disease (high blood pressure/diabetes etc.)	- Safely put dishes in and safely take dishes out of a hot oven (does not require ability to cook) - Uses oven safely to cook - Safely handles eggs, meat, seafood (refrigeration of foods, cleaning of cutting materials) - Washes fruits and vegetables - Uses oven mitts for hot items - Adjusts oven settings - Cleans greasy pans - Clean up and food storage
Wk.10	Stage 2 and 3: Small and Large Appliance Cooking	Sheet pan chicken with rice	Meal Production: Participants review oven and microwave safety in a peer-to-peer social setting. Emphasis on independence safely using large kitchen appliance, raw food handling. Attention on participant choice and preference with toppings and preferred assembly of dish. Carry over measuring and mixing from Stage 0 and knife skills from Stage 1. Staff supported with adaptive equipment, managing safety and attention to task.	Meal Planning/Grocery list	- Safely put dishes in and safely take dishes out of a hot oven (does not require ability to cook) - Uses a stovetop - Safely handles eggs, meat, seafood (refrigeration of foods, cleaning of cutting materials) - Determines when meat is fully cooked - Uses oven mitts for hot items - Adjusts oven settings - Cleans greasy pans - Clean up and food storage
Wk. 11	Stage 3: Large Appliance Cooking	Blueberry muffins	Meal Production: Participants review oven safety in a peer-to-peer social setting. Emphasis on independence with safely using large kitchen appliance, raw food handling. Attention on participant choice and preference for fruit mix ins. Carry over measuring and mixing from Stage 0 and knife skills from Stage 1. Staff supported with adaptive equipment, managing safety and attention to task. Participants showcase independent cooking skills with limited or time delayed prompting.	Plan Final Week Meal	- Stirs items in a mixing bowl - Puts leftovers away correctly (appropriate dished and wrapped/has lid) - Plans a weekly menu - creates shopping list for recipes - Uses oven mitts for hot items - Adjusts oven settings - Plans a meal that includes at least 3 dishes with fruits, vegetables, protein, etc. - Clean up and food storage
Wk. 12	Modified Stage 4: Introduction to Elective Cooking	Choose recipe from past 11 weeks to make independently	Participants execute choice and preference by choosing a recipe to create individually. Emphasis on preparing recipe by making a list, gathering ingredients, following recipe etc. Skills from previous 3 stages carry over. Participants showcase independent cooking skills with limited or time delayed prompting.	None	- Uses a recipe to gather at least 4 ingredients and 4 kitchen items to prepare a food dish - Follows recipes to prepare at least 4 simple food dishes - Follows at least 6 recipes to make cooked dishes that involve at least 3 ingredients - Clean up and food storage

a Added stage not originally apart of Goldschmidt and Song framework.

**Table 2:** Baseline demographics and anthropometrics of young adults with intellectual disabilities participating in a 12-week cooking skills and physical function intervention.

Demographic Variable	Overall N = 21
Age (yrs)^a^	22.2 ± 2.4
Sex	
Male	7 (33 %)
Female	14 (67 %)
Race	
White	12 (57.1 %)
Black	6 (28.6 %)
More than one race	3 (14.3 %)
Ethnicity	
Not Hispanic or Latino	17 (81 %)
Hispanic or Latino	4 (19 %)
ID Diagnosis	
Down syndrome	12 (57 %)
Autism	4 (19 %)
Other ID	5 (24 %)
Severity of ID	
Mild	13 (62 %)
Moderate	8 (38 %)
Living Situation	
At home with a parent	19 (91 %)
In a supported living environment	2 (9.5 %)
Weight (kg)	68.8 ± 20.9
Height (cm)	152.4 ± 13.1
Body Mass Index (kg/m^2^)	29.4 ± 7.2

a Values are mean ± standard deviation or sample size (n) and percent.

**Table 3:** Cooking skills, upper and lower body strength, aerobic capacity, and weight at baseline and post-intervention of young adults with ID who completed a 12-week cooking skills and physical function intervention.

	Baseline N = 20	Post-intervention N = 20	P-Value^a^
Cooking Skills: Skills not previously exposed to.	11.8 ± 11.1	6.9 ± 6.6	0.010
Cooking Skills: Skills completed independently	18.8 ± 12.4	21.6. ± 11.2	0.005
Cooking Skills: Skills completed with only a verbal prompt	30.6 ± 13.9	36.8 ± 9.8	0.010
Lower body strength (Leg Press, lbs)	124.3 ± 49.2	149.4 ± 53.1	0.003
Upper body strength (Grip Strength): Dominant Hand	20.0 ± 6.2	20.3 ± 5.5	0.672
Upper body strength (Grip Strength): Non-dominant Hand	18.6 ± 5.2	19.9 ± 5.4	0.076
Aerobic Capacity (2 Min Step Test, steps)	74.1 ± 25.4	71.0 ± 19.1	0.511
Weight (kg)	69.3 ± 21.3	68.2 ± 21.6	0.036

Note: Reported as Mean ± SD.

a Paired *t*-test.

## Data Availability

Deidentified individual participant data (including data dictionaries) will be made available, in addition to study protocols, the statistical analysis plan, and the informed consent form. The data will be made available upon publication to researchers who provide a methodologically sound proposal for use in achieving the goals of the approved proposal. Proposals should be submitted to the corresponding author at lptomey@kumc.edu.
